# Supplementary material for: Extremely Ultranarrow Linewidth Based on Low-Symmetry Al Nanoellipse Metasurface
Source: Nanomaterials (Basel). 2022 Dec 24;13(1):92. doi: 10.3390/nano13010092 (PMC9824327; doi:10.3390/nano13010092)
Supplement: Supplementary file 1 [file nanomaterials-13-00092-s001.zip › nanomaterials-2069042-supplementary.pdf]

nanomaterials

Extremely Ultranarrow Linewidth Based on Low-Symmetry Al Nanoellipse Metasurface

Liangyu Wang, Hong Li, Jie Zheng * and Ling Li *

Laboratory of Micro-Nano Optics, School of Physics and Electronic Engineering, Sichuan Normal University, Chengdu 610101, China
* Correspondence: zhengjie@sicnu.edu.cn (J.Z.); lingli70@aliyun.com (L.L.)

Effects of semi axis and film thickness of Al nanoellipse metasurface

We further performed the simulations of the following combinations: 1. Keeping the ratio of the semi major-axis to the semi minor-axis constant of 2, increase the semi major-axis from 100 nm to 150 nm (at 10 nm intervals); 2. Increase the *a*/*b* from 1 to 3. As depicted in Figure S1, when keeping the *a*/*b* constant, with the increase of the semi major and minor axis, the FWHM appears to be broader. And when comparing the three different ratios of the semi major and minor axis, the smaller the ratio, the narrower the linewidth. Therefore, the modulation of ultranarrow linewidth is closely related to the change of the semi minor-axis (*b*), but not the major-axis (*a*). This derives that the polarization direction is along the minor axis.

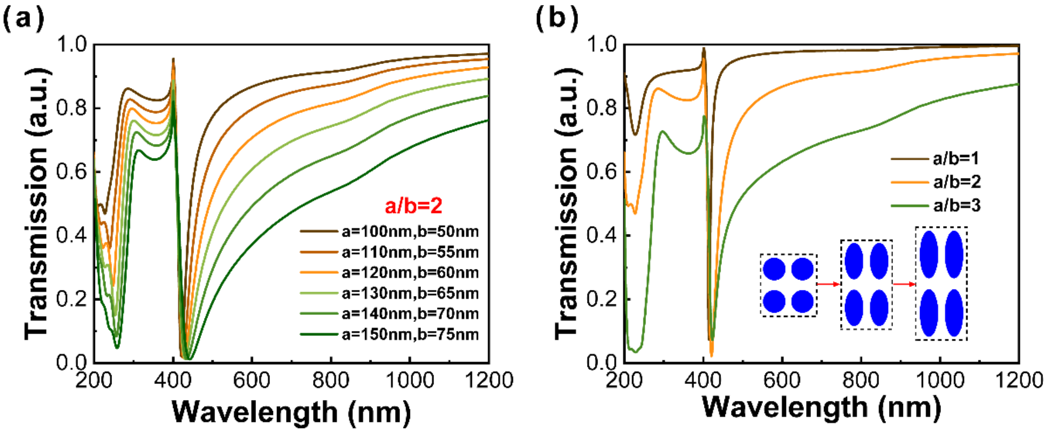

Figure S1. The transmission spectra of periodic arrays with varied (a) semi major- and minor-axis while keeping the *a*/*b* constant of 2. (b) a/b (a=b=40nm, a/b=1; a=100nm, b=50nm, a/b=2; a=180nm, b=60nm, a/b=3). (The small window shows the schematic illustration of varied structural parameters of and periodic arrays).

Simulation results demonstrate that the optimal configuration of the semi-major and -minor axis is determined as 40 nm and 20 nm, respectively. With the increasing of *d* from 60 nm to 100 nm, the corresponding reflectance spectra have been recorded. The influence of the height of the Al nanoellipse metasurface uncovers that the resonant modes are insensitivity for the variation of Al film thickness (see the Figure S2). We can intuitively observe that the linewidth is always about 27 nm with the increasing of *d*, as depicted in Fig.S2(b) from the enlarged view.

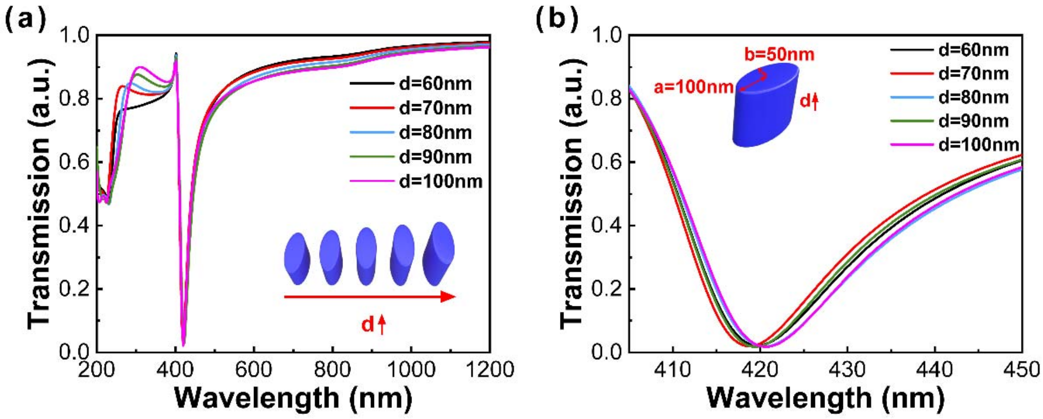

Figure S2. (a) Reflection spectra of Al nanoellipse metasurface with different thickness. (b) An enlarged view of Figure S2(a).
